# Dysentery

**Published:** 1823-06-01

**Authors:** 


### VIII.

### DYSENTERY.

1. *Medical Report of the Whitworth Hospital House of Industry; containing an Account of Dysentery, as it appeared in the latter end of* 1818. By J. Cheyne M.D. F.R.S. Ed. Physician General to his Majesty's Army in Ireland.

[Dublin Hospital Reports, Vol. III. Dublin, 1822.]

2. *Observations on the Acute and Chronic Dysentery of of Ireland; containing a Historical View of the Progress of the Disease in Ireland; with an Enquiry into its Causes; and an Account of its Symptoms and Mode of Treatment; with a Report of Selected Cases.* By John O'Brien, M.D. Fellow and Censor of the King and Queen's College of Physicians in Ireland; and Physician to the Fever-Hospital and House of Recovery, Cork and Dublin. 8vo. pp. 100, Dublin, 1822.

Faithful reports of epidemic diseases are very useful, as they enlarge the mind by acquainting us with the modifications produced by climate, modes of life, classes of society, and constitutions of the air. These reports *ought* to have the effect of guarding us against placing implicit confidence in a remedy because it has been successful in one epidemic—and of condemning it because it has not proved all-powerful in another. Yet the reverse of this salutary effect is not unfrequently experienced; such is the waywardness and weakness of the human mind.

It need not be stated that dysentery has proved a great scourge in our camps, fleets, and even in our cities, at times. In England, however, it has not prevailed to any great extent, in an epidemic form at least, for a good many years. Not so in the sister kingdom. In the course of the last five or six years, we are informed by Dr. O'Brien, this disease has been more frequent and fatal than usual in Dublin, and generally throughout Ireland. It was the precursor, and has continued the survivor of the late epidemic fever of that country. In its complications with the fever it aggravated that disease, and greatly augmented the mortality. It appears, from medical and historical records, that Ireland has been always considerably obnoxious to this form of disease. Sydenham entitles the flux to at least the second rank among the *leaguer* sicknesses of the 17th century in that unhappy country; and Walker, in his history of the siege of Londonderry, gives a lamentable account of the havoc produced by dysentery, both in the garrison and in James's army. Dr. Cheyne found, on examining the records of the Army Medical Board in Ireland, that dysentery has much decreased among the troops of that country within the last twenty years—doubtless on account of the great improvements in the barrack and hospital system. It appears from the same records, that the English and Scotch regiments generally suffered on their first coming to Ireland with this disease.

The patients admitted into the Whitworth Hospital, under Dr. Cheyne, "were frequently of worn-out constitutions," and had co-existent organic diseases, which, of course, rendered the cure more difficult. That it was, in the present instance, rather one of the forms of the prevailing epidemic fever, than genuine idiopathic dysentery, such as we meet with in the hotter regions of the earth, will appear probable from the following statement of Dr. Cheyne.

"Very often dysentery arose during convalescence from fever, in which case I several times ascertained that the preceding fever was not attended with any unusual gastric or enteric irritation. Sometimes the disease commenced at the termination of fever, and then it was at first so slight as to appear like a salutary crisis, its only symptoms being mucous stools with a degree of uneasiness at the termination of the rectum. Frequently the disease arose in the course of fever. Nausea, mucous or bilious vomiting, tenderness of the epigastrium, and deep yellow suffusion of the skin, characterized many fevers which occurred in the autumn of 1818; and when symptoms of dysentery took place before those of fever ceased, first, there were borborygmi and tormina, then mucous and bloody stools and tenesmus, the character of fever being gradually lost as that of inflammation of the mucous membrane of the great intestines was established; but from the peculiar nature of the fever of the season it was sometimes difficult to mark the precise time when the new disease commenced. The bilious fever of the autumn continued till near the termination of winter, consequently it existed as long as the dysentery was prevalent in the Hospitals of the House of Industry. When the symptoms of bilious or gastric fever were exchanged for those of dysentery, probably an extension took place of the irritation from the mucous membrane of the stomach and small intestines to that of the large intestines; hence borborygmi and tormina, as they occur in affections of both portions of the intestines, were not sufficient to mark the change in the disease till they were attended by mucous bloody stools, then indeed the case no longer admitted of doubt." 17.—*Dub. Hosp. Reports, Vol. III.*

Dr. Cheyne indeed says, that the disease was often *apparently* unconnected with the prevailing fever, and then it was assigned by the patients to cold, damp, hardships, &c. At page 19, he remarks that fever and dysentery were frequently converted one into the other; and that it seemed as if the contagion of fever, from some modifying condition of the system, "may have produced at one time fever, at another dysentery." These circumstances must be carefully remembered when we come to speak of the treatment; for it would be highly unfair, as well as unphilosophical, to expect the same results from particular remedies under the above circumstances, as where there was no epidemic constitution, no contagion, no fever, and no broken down constitutions.

"In almost every patient," says Dr. Cheyne, "of whose case we obtained a distinct account, whether the disease arose from fever, or was unconnected with it, a remarkably foul taste was complained of, such as often exists when there is a deficiency of bile in the intestines; considerable flatulence also existed, then rigor took place, which was the commencement of pyrexia: there was a case or two, in which the rigor took place after symptoms of dysentery were established. In a few cases nausea occurred as one of the earliest symptoms; in a few, tenderness of the belly; in several, a severe lancinating pain in some of the abdominal regions, generally in the umbilical, or even lower; but in the common course of things the rigor was followed by gripings, a frequent inclination to go to stool, with a desire to continue the effort, by which the bowels are emptied, and to these symptoms there was soon added a feeling as if the effort had been unproductive or incomplete,—as if there was still something remaining in the bowels; the skin was dry and hot, sometimes pungently so, the stools from the first were loose, perhaps not immediately undergoing any other change in their appearance, but in general they were mucous, and in rather more than one half of the cases there was a discharge of blood on the very first day of the disease." 20.

After a most diligent enquiry, they "could not discover that scybalæ were passed by any one of the patients." We wonder indeed how the idea ever could have been entertained that scybalæ were in any way connected with dysentery; for in nineteen cases out of twenty, no scybalæ will be seen, in any climate, or in any form of the disease. This has not been a harmless error. The search after these imaginary matters in the bowels, or rather the anxiety to dislodge them, has led to a cruel and injurious system of purgation in dysentery. In the discharged matters, we observe Dr. Cheyne enumerates almost every kind of secretion and excretion, healthy or morbid, except bile.

"In many respects," says Dr. Cheyne, "the dysentery of 1818 resembled that which has been described as occurring in tropical climates.* As in the latter variety, so in the former, the functions of the liver and skin were disordered from the commencement of the distemper, and continued thus until its termination. The skin was remarkably unyielding, and, if we may judge by the appearance of the stools, the biliary secretion was often suspended for many days."—Reports, p. 22.

Dr. Cheyne's prognosis was unfavourable in proportion to the degree of tenderness in the abdomen, pain, frequency of stools, fever, "and more especially quickness of pulse." It was very difficult to ascertain whether, and to what extent, there was ulceration of the mucous membrane. When the disease continued twelve or fourteen days, considerable emaciation usually came on; and when to this was added a haggard look, with quickness of pulse and abdominal tenderness, there was little hope of recovery.

"A harsh, dry, opaque, dirty looking skin, a florid, clean, varnished tongue, vigilance, a hollow eye, and pallid, wasted, faded cheek, a desire to go to stool immediately after drinking, pains in the knees, and cramps in the legs, fits of dyspnœa, a tendency to œdema and ascites, belong to a more advanced stage, but not to the last, which was characterised by extreme emaciation, supine posture, involuntary stools, a thin reddish secretion flowing without check, sordes on the teeth, hiccup, tendency to delirium, difficulty of swallowing, and a pulse like a thread."—Cheyne, p. 23.

---

* "Johnson, p. 354."

At one time a majority of Dr. Cheyne's patients died, who had been ill more than six or seven days, and of those who were emaciated scarcely one recovered."

"Dysentery was productive of several forms of dropsy,—ascites, and anasarca in particular; and it is worthy of remark, that a swelling occurred in several of the patients, both males and females, resembling the phlegmasia dolens in all respects but in its connexion with parturition. In one or two cases symptoms of hydrothorax were observed."—Rep. 25.

There was sometimes a translation of disease to the lungs, with a degree of effusion in the cavity of the pleura, and blood in the air cells, evinced by oppressed respiration, pain in the chest, a short teazing cough, scanty mucous expectoration, constant jactitation, small pulse, colliquative perspirations, and great prostration of strength.

The dissections made at the Whitworth Hospital presented the diseased appearances "chiefly in their commencement, or after the morbid action had run its course." Death scarcely ever took place while the disease was in its intermediate stages. The ascertaining of the symptoms therefore which belong to the commencement of the ulcerative process must be ascertained by succeeding pathologists.

"The mucous membrane of the stomach, and small intestines, sometimes presented an inflamed appearance, which, in general, became more remarkable as we approached to the great intestines; then ulceration began to show itself, at first superficial, afterwards laying bare the muscular fibres of the intestines; the ulcerations became larger, more numerous, and deep, as the rectum was approached; but it was remarked that the last three or four inches of the rectum were sometimes pretty sound. The peritoneum was found less diseased than might have been expected." 29.

The deaths which took place in an early stage of dysentery, were from fever or some other fatal disease. When this was the case, "a peculiar state of the mucous membrane of the stomach and intestines was noted."

"This membrane was found of a more or less deep red or purple colour; it was rather thickened, soft and pulpy; the least violence destroyed the continuity of the surface, which had lost its healthy smoothness, was uneven, and not unfrequently rough and granulated, the blood vessels being enlarged and loaded with blood: where the alteration in texture was inconsiderable, there were numerous spots of a deep red colour, which at first resembled extravasations, but, on close examination, seemed to be blood vessels, ramifying in an arborescent form, and connected with veins. The stomach contained a viscid mucus, firmly adhering to its coats, and mixed with an opake yellow, or whitish matter; the contents of the large intestines were fluid, and of a yellowish green colour."—Cheyne, p. 29.

Dr. Cheyne relates an interesting case of complicated fever and pneumonia, where dysentery supervened four days before the patient's death.

"She had a lancinating pain in the abdomen, tormina and discharge of blood by stool; symptoms, which were relieved by castor oil, followed by compound powder of ipecacuanha. The most interesting part of the dissection was the display of a highly vascular state of the whole of the mucous membrane of the intestines, without ulceration or erosion; proving, that a free discharge of blood may from this, as from other mucous surfaces, take place without previous ulceration or erosion." 30.

Three or four other cases, presenting *nearly* similar appearances, are related; and they are extremely valuable, since dissections in the early stages of dysentery are very rare, for very obvious reasons. It is only indeed during epidemics like this, where dysentery forms one of its complications, that such dissections can be got at. Dr. Cheyne caused preparations to be made from the intestines of dysenteric patients, and these are preserved in the Whitworth Hospital. They may be divided into two classes—those in which the intestine is thickened, and those in which it is not. In the first class may be seen specimens of the mucous membrane increased in vascularity, without abrasion or ulceration—the same membrane covered with coagulable lymph—the membrane simply abraded, its epidermoid coat removed—the membrane ulcerated, the intervening portions natural—and, lastly, the mucous membrane partly ulcerated, partly covered with coagulable lymph. In the second class, where there is no thickening, we have the following varieties. The mucous membrane simply abraded—the same ulcerated, the intervening spaces natural—the mucous membrane rugous and ulcerated—the same ulcerated and filamentous, hanging in shreds as if sphacelated—the membrane partly ulcerated, partly removed, exposing the muscular coat. "In many of these preparations the mucous membrane, when not eroded or ulcerated, is covered with an exudation of coagulable lymph."

In a majority of cases the liver was apparently sound in structure; but in a good many instances remarkably otherwise. "In a considerable number of bodies it was in a state of great sanguineous congestion."

Dr. Cheyne properly remarks, that it is a great error to suppose that a disease admits of only one mode of treatment. In the course of the season, the practice of the Whitworth Hospital underwent several changes—their confidence in certain methods of cure fluctuated, being sometimes weakened by experience, "and then restored by experience still more extensive."

"For example, the treatment by means of mercury did not succeed so generally as I expected. Calomel and ipecacuan; the blue pill and ipecacuan; doses of five grains of calomel and half a grain of opium, at stated intervals of four or six hours; larger doses of the same medicines, a scruple of calomel and two grains of opium—all these methods often failed when the disease was established, not only with me, but with some of my colleagues, who treated the disease in the fever wards; and yet I have no doubt that the mercurial treatment is entitled to confidence, although probably not to the same degree of confidence that it is in other climates." 41.

We recommend the following passage to the serious consideration of those of our brethren who have not witnessed dysentery on a large scale.

"The doctrine pretty generally taught with respect to the nature and treatment of dysentery did not apply to the form of that disease, which is under consideration. We are told that, before dysentery has long continued, the fæces are formed into compacted masses, and that the patient complains of a load in his intestines, with which they feel oppressed. The only complaint of this kind, which I heard, was of a sensation, as if the lower part of the intestines had not fully discharged its contents, producing tenesmus. We are directed to give purgatives, until the load felt by the patient shall have been removed, and we are told that purgatives have not performed their office until, by removing scybala, the cause of this sensation is removed, a rule which it is plain cannot apply to a disease, in which there are no scybala. Indeed that theory of the disease, which supposes the symptoms of dysentery to depend upon an accumulation of indurated fæces, will require to be reconsidered, when perhaps it will appear in this, as in other cases, that one of the effects of disease has been mistaken for its cause. In many instances purgative medicines seemed greatly to increase all the sufferings of the patient, and castor oil, the cathartic to which a preference is usually given, frequently roused the dormant griping pain, so that it appeared that its right to be preferred in this disease, now almost prescriptive, had been too hastily conceded. Finally, we are often cautioned against venesection, which was certainly the remedy the least equivocal in its effects, the most uniformly useful of any which we employed in the Whitworth Hospital." 42.

Of the cases not attended with much fever or pain, and which were admitted during the first few days of the disease, a purgative early in the morning, ten grains of Dover's powder early in the afternoon, and the same at bed-time, with low diet, restored many to health. "But in several of these cases the disease *spontaneously ended*, as the concurrent fever often did, in free perspiration." This consideration, as our author wisely observes, was calculated to "reduce reliance on this plan within just limits." When the disease assumed a more inflexible character, and was attended with considerable pyrexia, tenderness of the abdomen, muco-sanguineous stools, tormina, and tenesmus, our author would prefer to the mercurial treatment, the old proceeding of venesection, saline purgatives, warm bath in the evening, and lastly an anodyne diaphoretic at bed-time.

When with pyrexia, the stools consisted chiefly of bloody mucus, and the abdomen was very tender, blood-letting was never omitted, and the symptoms were greatly mitigated thereby.

"But there were cases requiring a procedure still more decided; cases wherein the disease had resisted the foregoing method so long as to excite an apprehension of extensive ulceration, or in which it began with unusual violence, or in which, after proceeding moderately for a time, symptoms of great danger suddenly arose; as for instance, intolerance of the slightest pressure, agonizing pain, unceasing tenesmus, great pyrexia; in these cases the method recommended by some of the late writers on tropical diseases* was used with advantage. In the course of a single hour the patient has been largely blooded, has taken two grains of opium and a scruple of calomel, has had the warm bath, and been swathed in flannel,† by which means we have obtained breathing time to pursue our plans more deliberately. Were the same cases again to be placed under my care, I would not hesitate to prescribe opium in doses of four or five grains, as it was the opium chiefly which seemed to me to arrest the progress of the inflammation, and whatever in such a case procured respite for the patient from agony, sometimes proved of permanent benefit. After the large dose of calomel and opium, calomel in doses of five grains, with half a grain of opium, every third or fourth hour, was given till it produced ptyalism; as soon as the mercurial influence was apparent from the state of the mouth, the mercurial was laid aside, and a mixture with balsam of capivi‡ was given every fourth or sixth hour, from which the greatest relief was often obtained; the fæces, from being of a bottle green, and mixed with mucus (the effect of the calomel and opium) and passed with tenesmus, became more natural in appearance, and were voided less frequently; the patient considered himself cured, and in many cases his recovery followed, although not without interruptions, requiring various changes in the form of the anodyne; such changes, it may be observed, were generally productive of benefit; thus the chalk julep, or catechu mixture, with laudanum, when the capivi draught lost its effect, were substituted. It results from a consideration of the cases in my possession that venesection, calomel, and opium, followed by the capivi mixture, with farinaceous diet, proved more

---

* See Dr. Johnson's work. † See Mr. Dewar's work.

‡ See Dr. Pemberton's work.

successful than any other method which was adopted in the severest cases; yet it often lamentably failed, of which sufficient evidence will be found in the table of unsuccessful cases. It ought not to be forgotten, that when the skin was restored to a natural state by the mercury, the case generally proceeded favourably." 45.

Although mercury failed in many cases, and was, as might be expected, injurious in the ulcerative stages of the disease, yet venesection was beneficial not only at the beginning of the disease, but even after it was supposed that ulceration had commenced.

At one period in the Whitworth Hospital, when the medical officers began to lose confidence in every measure, not even excepting the mercurial treatment, a physician proposed cream of tartar as a remedy that would cure all cases of the disease. Although little was expected from such a measure, yet, in such desperate circumstances, it was fairly and fully tried—by the physician himself. The dose to an adult was half an ounce of the powder finely levigated every fourth or sixth hour. It was soon discovered that this medicine "failed in many instances," but it was admitted "that some patients who took it were restored to health, who, Dr. C. thinks, would have sunk under any of the modes of treatment then in use." The "white powders," therefore, became rather a favourite in the hospital thenceforward. In some it occasioned much distress, and several individuals refused to take it. "But when persevered in, it often brought down bile, and then by giving it only once or twice in the day, and alternating with it Dover's powder, the capivi, or chalk mixture, and using the baths, the cure was completed." We have remarked that, in almost all cases of dysentery, the *function* of the liver is torpid or impeded—at least, the biliary secretion appears exceedingly scanty. This, we conceive, is an important feature in the pathology of the disease, and was not till lately known, or at least promulgated. We are happy in having the corroborative testimony of such a man as Dr. Cheyne on this point.

"In the property of bringing down bile, I think it must be admitted that the cream of tartar excels most other purgative medicines, and in this way its efficacy in dysentery may probably be explained, for that the suspension of the biliary discharge is a very important part of the disease few will deny, who have seen the great relief which sometimes, in this disease, follows a discharge of bile." 49.

Castor oil, so generally beneficial in idiopathic dysentery of the hotter climates, did not, as was before observed, agree with patients in the epidemic under review. But when half an ounce of the oil was combined with ten or twelve drops of laudanum, the combination was found to be an invaluable medicine. When castor oil also was combined with the rectified oil of turpentine, it operated with less pain than when given without the latter medicine. "This, which is also a valuable combination, borrowed from puerperal practice, and much used in Dublin, sometimes effectually reduced that tumefaction of the belly which often so much distressed the patient." Injections, composed of superacetate of lead and opium, in solution, ranked high in the Whitworth Hospital. Dr. Barker has given, with success, this combination internally in dysentery. For the cases and dissections, thirty in number, we must refer to the volume itself.

When we consider that the disease in question was complicated with, indeed, we might say, it was a *form* of, the the prevailing epidemic fever—that it occurred among the lower orders of Dublin, notorious for squalid poverty, filth, and intemperance, we ought not to wonder at the occasional want of success attending some measures found highly efficacious under other circumstances—especially among men in the prime of life, in our army and navy, where the disease was instantly recognized on its first appearance, and treated vigorously. Still, with all the disadvantages attendant on Dr. Cheyne's patients, his report goes, in the main, to confirm what has been inculcated by our naval and military practitioners, during the last ten or fifteen years, in respect to dysentery. The interrupted or defective function of the liver, the corresponding state of the skin, the non-appearance of scybalæ, the amelioration produced by an increase of the biliary secretion, the safety and utility of bleeding, and the general efficacy of opium and mercury, are all substantiated by the said report. All we wonder at is, that the measures did not more frequently fail, under such circumstances, than actually happened.

Dr. Cheyne has appended a supplement, containing an account of dysentery, as it appeared in the 79th regiment, at Limerick, in the year 1821, drawn up by Dr. Perston, assistant surgeon of that corps. The prominent feature in this dysentery was the following:—

"The liver was invariably deeply engaged in disease; in general considerably enlarged; and its whole structure apparently destroyed. In two cases the surface was studded with large yellowish white spots, which, on cutting into them, were found to contain pus, and yellow purulent matter was found in distinct cells throughout its whole extent. The concave surface commonly presented a livid aspect, and was indurated. The gall bladder usually nearly empty." 89.

Dr. Perston used active depletory means, and "in all the severe forms of the disease, after premising general evacuations, and when the inflammation was checked, mercurial frictions were ordered over the region of the liver." "I was led," says Dr. P. "to try the effects of the remedy, in consequence of having witnessed that the structure of that viscus was more or less destroyed in every fatal case of this disease, though it had been specially remarked, that no feeling of pain—not even a sense of weight or uneasiness had been referred to that organ by the patient." Although, in several instances, our author attributes the recovery of his patients to these frictions, yet he did not find them so extensively beneficial as he had anticipated. We wonder he saved a single patient; for where the biliary organ was so generally implicated in structure as in this dysentery, no time should have been lost, *from the very beginning*, in the introduction of mercury by the mouth as well as by the skin. It is thus that remedies are frequently injured in reputation by the injudicious modes of their administration! We cannot take leave of Dr. Cheyne without expressing our great esteem for his candour, zeal, and ability in the prosecution of all his professional researches.

We now come to the second work at the head of the article. Dr. O'Brien has gone more formally to work than was necessary, by inquiring into the definition, history, and ancient literature of dysentery. There was surely no need for this, in order to give the public an account of a recent epidemic. But too many writers fall into this practice. Being rather undecided as to contagion, Dr. O'Brien thinks that Cullen's definition ought to have "aliquando" introduced before the adjective "contagiosa," which would render it, he thinks, unobjectionable. Our author, however, has never seen any instance where the disease was unequivocally propagated by contagion. He supposes it possible that when the disease is epidemic in camps, ships, crowded hospitals, or populous cities, "the attendant fever may assume a malignant and typhoid character, and the disease become contagious." To us it appears evident that *dysentery* is not contagious; but that *fever*, having a determination to the *bowels*, may be so, the same as a fever with a determination to the lungs or brain.

In respect to the *causes* of the late epidemic dysentery in Ireland, our author has reason to consider them the same as those which gave origin to the epidemic fever. The seasons were wet and inclement, the crops had failed—and hunger and wretchedness prevailed. The consequence was disease in various shapes.

"In this state of things the dysentery appeared in Dublin, in 1817, and has since continued, with some short intermissions, to maintain its ground, to an extent unknown for the last fifty years. The predisposing and exciting causes are clearly pointed out, in the extraordinary combination of physical evils above described. The visible agents in operation, during the prevalence of this malady, were famine, and want of every description, which, probably, operated as predisposing causes; and secondly, cold, excessive moisture, and unwholesome food, which appear to have been the exciting causes." *O'Brien*, p. 18.

Our author states that he has not been able to trace that connexion between dysentery and disease of the liver so much insisted on by some writers on tropical diseases. Dr. O'Brien is led into the same misapprehension that many others have been led into, by confounding disordered *function* with diseased *structure*. The best writers on tropical dysentery have observed *impaired function* of the liver in the dysentery of hot climates, and we appeal to Cheyne's and to Ferston's papers just reviewed, for a corroboration of the same in the dysentery of Ireland. Our author offers a curious reason why the liver is not concerned in dysentery—"neither are the discharges tinged with bile, but, on the contrary, the disease seems to be attended with an opposite condition of the liver, or *a deficient secretion* of that fluid." If this be not functional disorder of the biliary organ, we know not what is. It is the precise functional disorder too which is insisted on as obtaining in tropical dysentery, so much has Dr. O'Brien misunderstood the writers in question. He has himself supplied the proof of their accuracy of observation. Into another error has Dr. O'Brien fallen, namely, that cholera is produced by a redundant secretion of bile;—an error that has been abundantly exposed of late years, as our readers well know.

"The salutary agency of mercury, in dysentery, seems to have strengthened the theory of its hepatic origin. But, it must be recollected that mercury exercises a powerful influence over the cutaneous and mucous surfaces also, restoring them to their healthy state, with a more rapid and evident agency than perhaps any other medicine: its power in the cure of cutaneous diseases, and in the inflammation of the mucous membrane of the larynx, and, in fine, its general action as a diaphoretic, sufficiently prove its direct action on those surfaces." *O'Brien*, 22.

As to structural disease of the liver, even that was pretty frequently observed in the late epidemic, according to our author's own observations. The *facts* being thus ascertained, we have no objection to our author's *explanation*, as thus propounded:—

"There is no doubt, however, of the co-existence of dysentery and disease of the liver in a multitude of instances; and the most rational explanation which suggests itself of the reciprocal influence of the two diseases on each other seems to be the following:—Persons labouring under previous disease of the liver, become affected with debility and turgescence of the mucous coat of the intestines, in consequence of an impeded and repelled circulation in the diseased liver, which more readily disposes them to fall into the dysenteric state, on the application of the exciting causes formerly mentioned. In such cases disease of the liver must be considered merely as a pre-disposing cause; and that it is frequently one there can be no doubt." 22.

The remote causes, Dr. O'Brien, in common with the best observers, attributes to "sudden variations of temperature," acting on organs predisposed to disease by intemperance and unwholesome food. The occult state of the air, so often mentioned but never yet explained, our author does not deny, but thinks we had better leave this epidemic constitution out of the question, and confine ourselves to those causes that are obvious to the senses. To this it may be objected, that the obvious causes are not always adequate to the explanation, and then the mind reverts necessarily to some hidden agency.

"However authors have differed concerning the remote causes of dysentery, there seem no grounds for doubting that its proximate cause is inflammation of various degrees of intensity, affecting the mucous membrane of the intestines, chiefly the large intestines. This fact has been amply proved by dissection, by which either inflammation or some of its sequelæ, as stricture, ulceration or thickening of the mucous membrane have been invariably detected. A close analogy may be traced between catarrh and dysentery, and the production of both diseases may be accounted for in a similar manner. It is a long established principle in pathology, that all those causes which produce a suppression of the cutaneous perspiration are capable of generating catarrh. The principal of them is cold, by which the perspiration from the skin being checked, is thrown inwardly on the mucous membrane. It must be remembered that the skin and mucous membrane are continuous surfaces, and readily sympathise with each other. Hence the rapid translation of the excretions from one surface to the other, and their reciprocal action in a state of disease. The afflux of blood to the mucous membrane produces in that organ an inflammation of greater or less activity, according to the violence of the exciting causes, and thus the phenomena of catarrh arise. On the same principle may the phenomena of dysentery be explained, making allowance for the different nature and uses of the organ affected in this latter disease. It has been generally observed by practical writers, that the period most favourable for the production of dysentery is when a cold moist autumn succeeds a warm dry summer. Under such circumstances, it is probable that the skin acquires, from excess of heat and transpiration, a morbid state, which renders it more sensible to the succeeding winter's cold. If cold be then applied primarily to the lower part of the body, we can readily trace the connexion between cause and effect, and the mode of operation, if dysentery be the consequence.

"When, under such circumstances, some important viscus, as the liver, happen to be diseased, and its circulation obstructed, the passage of the blood through the vena portæ is impeded, and that fluid thrown back on the mesenteric veins and arteries, causes a kind of habitual turgescence, or sub-inflammation in those vessels. If, in this state of things, the cutaneous perspiration be interrupted by cold applied to the abdominal region, the balance of the circulation will be still further deranged. The circulation will diminish on the surface, and increase in the cavities, and by a uniform law of the animal economy, the loss of the healthy discharge on the surface will be compensated by a morbid one in the interior. If the serous membrane be the weaker organ, dropsy may be the result; if the mucous, dysentery." 30.

Dr. O'Brien here takes an opportunity of differing from Dr. Johnson in respect to the proximate cause of dysentery. There is not, however, so much real difference of opinion between them as Dr. O'Brien imagines. Dr. Johnson does not deny that a state of inflammation is an early condition in dysentery, but he contends that inflammation is not the *first* link in the chain of morbid action. He maintains, for instance, that the functions of the liver, and glandular viscera of the abdomen are disturbed—that the balance of the circulation and of the excitability is next thrown on the mucous membrane of the intestines, where a state of *irritation* first obtains, and if that be not quickly removed, a state of inflammation, and ultimately ulceration ensues. This attempt of Dr. Johnson's to trace out those phenomena of dysentery which precede actual inflammation has been, by several, misconstrued into a denial of the existence of inflammation in the disease. Certainly it is Dr. Johnson's belief that the inflammation is a sequence or consequence of other previous morbid states, irritation for instance, and therefore can hardly be called with strict propriety the proximate cause of the disease. As, however, it universally obtains when the disease has become well established, there is no objection, in a practical point of view, to consider inflammation of the mucous membrane as its proximate cause.

Dr. O'Brien's description of the symptoms of dysentery need not be entered on here. We observe that he still believes in the existence of scybalæ, in dysentery, though he acknowledges that they cannot be considered in the light of one of the causes of the disease, and that he has never observed them on dissection. That indurated fæces may happen to be lodging in the cells of the colon when dysentery attacks a patient, and that they may be dislodged and discharged in the course of the disease, there can be no manner of doubt; but that to this accidental circumstance should be attached any importance, we are inclined to deny.

From the principles laid down by our author, (the inflammatory,) it follows, of course, that the antiphlogistic treatment must form the basis of the cure.

"In the execution of this plan the two great objects which it is necessary to keep in view are—to restore the functions of the skin, and to unload the mesenteric system of its excess of blood. That blood-letting is a remedy calculated to fulfil these indications, but particularly the latter, there can be no doubt; in the first stage of dysentery I conceive it may be administered with as much confidence, and to as great an extent as in most of the phlegmasiæ. In almost all the cases of dysentery which I had an opportunity of treating in the early stages of the disease, within the last three or four years, I have employed venesection freely, and have uniformly witnessed its good effects in mitigating the tormina, lessening the frequency of the discharges, and allaying febrile excitement." 43.

Where general bleeding dare not be carried farther, the local bleeding by leeches is recommended. It is rather curious that, if dysentery consist in neither more nor less than inflammation of the mucous membrane of the intestines, venesection should not be able to cure it, as it unquestionably has cured many other phlogoses. Let us us hear Dr. O'Brien's sentiments.

"By the opinion which I have stated in favour of this remedy, I by no means wish to set it up as an infallible cure for this disease; on the contrary, I much doubt if it has ever succeeded by itself, or if it is capable of succeeding in curing the disease, *as it often succeeds in others of the phlegmasiæ.* It appears to me that blood-letting ought to be considered only in the light of a useful auxiliary to the other modes of depletion hereafter to be mentioned, and as applicable principally, if not solely, at least in its general form, to the early stages of the disease." 44.

The next remedies on our author's list are "sudorifics and purgatives combined with opium."

"The mercurial salts, James's powders, and Dover's powders, are the principal medicines of this class applicable to the cure of dysentery: four or five grains of calomel, with as much James's powder, and a grain of opium, may be given every third or fourth hour, or even more frequently, according to the exigence of the case. This plan being pursued for the first twenty-four hours of the curation, ought to be succeeded in the next twenty-four by the purgative plan. With respect to this, however, no fixed rule can be laid down; in very urgent cases I would prefer continuing the medicines above-mentioned, and exciting the mercurial action as rapidly as possible, rather than waiting for the more doubtful and tardy benefit to be derived from purgatives. The purgative which I most frequently employ is the old fashioned one of castor oil and tincture of rhubarb. This purgative is certainly preferred by most patients, and appears to operate with less irritation than the saline purgatives. Occasionally a few drops of the tincture of opium are added to lessen its irritation." 45.

The author we believe to be correct in asserting that the combination of calomel and opium is inferior in efficacy to that into which antimonial powder enters. The latter combination, viz. "calomel, opium, and antimonial powder," Dr. O'Brien "has no hesitation in pronouncing the most powerful anti-dysenteric remedy we possess in the dysentery of this country;" and, he might have added, of most other countries too. Speaking of the various specifics which have been lauded in dysentery, our author remarks—

"The truth is, no single medicine deserves the name of a specific in this disease; but, of all known remedies, mercury certainly approaches nearest to that character, and possesses the most decided controul over this disease. Its beneficial agency appears to be exerted in restoring the functions of the liver, and promoting the mesenteric circulation in the first instance, and secondly, in restoring the healthy action of the skin itself. That it is adequate to effect important changes in the skin, will readily appear when we recollect its powerful influence in certain cutaneous diseases. In many instances it exerts its salutary agency before it reaches the point of salivation; in others the signs of amendment are delayed until it has arrived at this point, immediately after which, the dysenteric symptoms subside and convalescence commences." 47.

It is hardly necessary to remark that even this remedy will not always be successful, in all times, places, and stages of the disease. "I think," says Dr. O'Brien, "I may venture to affirm, however, that, if timely administered, it will succeed in a very great majority of cases. The failures which take place in this medicine, arise partly from the late period of the disease at which it is administered—a circumstance which must frequently occur in hospital practice."

Dr. O'Brien conceives that emetics have *undeservedly* fallen into disuse since the mercurial plan has become fashionable. This, we think, is true. Emetics are very powerful means of determining to the surface, and restoring the lost balance of the circulation. Sudorifics are universally allowed to be important remedies in this disease. The warm bath is obviously indicated. Our author makes some judicious observations on flannel bandages, counter-irritants, opiate injections, the mineral acids, &c. for which we must refer to the work itself.

*Chronic Dysentery.* Under this head Dr. O'Brien includes all those cases protracted beyond a duration of five or six weeks, and which are generally unaccompanied by fever, unless of the hectic kind. This form of the disease, which is usually, but not always, a sequel of the acute, is the more intractable of the two, and by far the most frequently met with in hospital practice. "The most numerous sufferers, indeed, from chronic dysentery, are the aged and infirm who have previously suffered from diseases of the liver and other abdominal viscera."

> "The symptoms of chronic dysentery vary in degree, according to the violence of the previous disease, and the extent of the structural injury sustained by the intestines from a simple relaxed state of the bowels, producing diarrhœa to a permanent ulceration, continually pouring forth purulent matter. In the milder cases the least exposure to wet and cold, or the slightest errors in diet, are succeeded by a diarrhœa, accompanied by gripes and tenesmus. In the more severe cases, the causes just mentioned are succeeded by a flux nearly constant, at least recurring at short intervals, of a fetid sanious matter, resembling the washings of raw flesh. On inspecting the discharges in those cases attentively, streaks of pus are evidently discernible on the surface of the ichorous matter discharged, which contains little of the substance, or even smell of natural fæces. In some cases the discharges are as black as pitch, in others they are in the opposite extreme of a dirty white colour, resembling the coagulated mucus of the nose, or dirty cream. This last appearance is characteristic of the last stage of very protracted dysenteries." 58.

This deplorable state will sometimes last for months, the patient gradually wasting away as in the last stage of phthisis pulmonalis—indeed, this disease might almost be termed "phthisis intestinalis." The appetite generally continues, and is sometimes ravenous; but indulgence in food always aggravates the complaint. In most cases where the disease had continued long, in our author's experience, the liver was found affected extensively.

*Morbid Anatomy of Dysentery.* The structural derangements found on dissection have been, of course, the products of inflammation, and inculcate the important lesson that our exertions should be directed to the prevention or subjugation of this state. In all the cases which Dr. O'Brien examined, one or other of the following appearances presented—ulceration or abrasion of the mucous membrane of the intestines—the colon, in every instance, thicker and more contracted than natural through its whole extent, but particularly at its lower flexure, where it bends over the ilium to form the rectum. Our author did not find the small intestines ulcerated, "but patches of a deep red colour were generally discovered here and there, especially on the ileum."

"In one case, the whole of the intestines presented a compact mass of adhesions, the small intestines adhering, in several places, to the peritoneum. In this case, the transverse arch of the colon, the duodenum, and the stomach, were firmly united together by adhesions. In this case also, the liver, the middle lobe of which extended two inches below the ensiform cartilage, was converted into a mass of tubercles of the large kind." 63.

In a majority of instances the liver was unaffected, the large intestines being the seat of the disease. The gall-bladder was always found distended with deep brown or dark yellow bile. "The mucous membrane (of the large intestines) was either obliterated or converted into a pulpy, bloody slime, which on being scraped off, presented an uneven surface, studded with small indurations, which no doubt were the mucous glands."

*Treatment of Chronic Dysentery.* The most essential part of this consists undoubtedly in dietetics, which ought to be of the mildest and most bland description. The experience of our author verifies that of Pringle, in respect to the utility of a milk diet. "The patient labouring under chronic dysentery should be placed altogether on a diet of milk and vegetable mucilage, as rice, arrow-root, sago, salep, &c." Flannels next the skin are absolutely necessary—the warm bath twice a week, using the flesh brush afterwards—a change to a warmer climate, if the patient live in this country; and to a colder, if a resident between the tropics.

*Treatment.* Where the patient is able to bear the operation of an emetic, at the beginning, Dr. O'Brien thinks it advisable to administer one: but if contra-indicated, then the bowels are to be unloaded of their acrid contents by gentle laxatives.

"A draught composed of castor oil, tincture of rhubarb, balsam of Copaiba, and a few drops of tincture of opium, has been generally directed by me under those circumstances; the purgative being repeated at longer or shorter intervals, as the case might seem to require." 71.

Dr. O'Brien's principal efforts have been directed to restore the functions of the skin and the abdominal viscera. With this view, sudorifics, combined with opium and a gentle mercurial alterative, were exhibited.

"The benefit of a slight alterative course of mercury will be apparent, when it is recollected how frequently disease of the liver is combined with inflammation and ulceration of the internal surface of the intestines in chronic dysentery. The former materially aggravates the latter, and forms a part of the general disease. Every measure, therefore, which tends to produce a healthy biliary secretion very considerably forwards the cure." 71.

These anodyne mercurial diaphoretics being continued a few days, are omitted, and alternated with astringents. "In slight cases, the mistura cretæ, with tincture of opium and tincture of catechu or kino (the common astringent mixture of hospitals,) answers very well." In severer cases, our author has used a solution of alum and sulphate of zinc, in cinnamon water, with tincture of opium. Oil of turpentine was tried, without producing any good effect. The patients described it as producing racking pains in the bowels, and giving no respite from the disease. The balsam copaiba, however, appears to have proved a valuable remedy; and the best medicine for correcting its nauseous taste was found to be milk, with which it mixes by means of sugar. If a few drops of any essential oil be added, the mixture is rendered still less disagreeable. The superacetate of lead did not succeed in our author's hands. The arsenical solution in two or three cases seemed to act as a powerful tonic to the system. In the following passage we agree with our author, and can vouch, from some experience, for its truth.

"Of all the remedies which have been employed for the cure of chronic dysentery, perhaps opium is the most universally remedial, and that which may be relied on with most certainty, as affording relief to the sufferers from this disease. In most of the anti-dysenteric compounds of former and modern times, it makes an important ingredient, and is the only efficacious one in many of them. In many instances there is little doubt but it has effected a cure, and in those where it has failed, it has considerably prolonged life, or soothed the bed of death." 75.

For the selected cases and dissections we must refer to Dr. O'Brien's work.

We may just say a few words on chronic dysentery before we close this article. We have seen a good deal of the disease, and we have personally felt it. The main pillar of the treatment is *rigid abstinence*. In nine cases out of ten, where such organic disease has not taken place as renders all remedies abortive, we verily believe that the patient might cure himself of chronic dysentery by a resolute system of starvation and perfect quiteude. If a man were to live on one small meal of farinaceous food in the day, and keep to his bed, he would do more than medicine can effect, without this precaution. But medicine may be of great assistance. A couple of grains of blue pill every night, with three or four of antimonial powder, and one or two of opium, will be very serviceable, taking a spoonful of the cretaceous mixture with opium or aromatics, *post singulam sedem liquidam*, for this is the only rule that can be laid down. Astringents we have often employed—and often with advantage; but if there be any hepatic disease, we would advise caution in their exhibition.

We have to return thanks to Drs. Cheyne and O'Brien, for their valuable and candid observations. We have enabled our brethren to judge of their merits by the ample analysis now laid before them.

